# Supplementary material for: Impacts of Postoperative Adjuvant Therapies on the Survival of Women with High-Risk Early-Stage Endometrial Cancer: A Cohort Study
Source: Cancers (Basel). 2025 Jan 8;17(2):187. doi: 10.3390/cancers17020187 (PMC11764345; doi:10.3390/cancers17020187)
Supplement: Supplementary file 1 [file cancers-17-00187-s001.zip › Table S1.pdf]

Supplemental Table S1. Postoperative VTE prophylaxis and treatment methods used in women with high-risk EEC.

	Prophylaxis (n = 515)	Treatments (n = 48)
UFH	269	5
LMWH	192	8
Fondaparinux	0	0
Warfarin	16	12
DOAC	38	16
Thrombectomy	0	0
Thrombolysis	0	1
IVC filter	0	5

DOAC, direct oral anticoagulants; EEC, early-stage endometrial cancer; HIRA, health insurance review & assessment Service; IVC, Inferior Vena Cava; LMWH, low molecular weight heparin; UFH, unfractionated heparin; VTE, venous thromboembolism.
All values are expressed as number.
