# Supplementary material for: Impacts of Postoperative Adjuvant Therapies on the Survival of Women with High-Risk Early-Stage Endometrial Cancer: A Cohort Study
Source: Cancers (Basel). 2025 Jan 8;17(2):187. doi: 10.3390/cancers17020187 (PMC11764345; doi:10.3390/cancers17020187)
Supplement: Supplementary file 1 [file cancers-17-00187-s001.zip › Table S2.pdf]

Supplemental Table S2. Characteristics of 18 women who received adjuvant chemoradiotherapy.

Adjuvant chemoradiotherapy	Case	Histologic types	Chemotherapeutic agents
CCRT	2		
EBRT ± VB	1	Carcinosarcoma	Ifosfamide
BV alone	1	Carcinosarcoma	Ifosfamide
Sequential therapy			
Chemotherapy after radiotherapy	8		
EBRT ± VB	6		
		Endometrioid adenocarcinoma	Ifosfamide
		Endometrioid adenocarcinoma	Ifosfamide
		Endometrioid adenocarcinoma	Ifosfamide
		Endometrioid adenocarcinoma	Ifosfamide
		Endometrioid adenocarcinoma	Docetaxel
		Endometrioid adenocarcinoma	Doxorubicin hydrochloride
VB alone	2		
		Serous adenocarcinoma	Doxorubicin hydrochloride
		Carcinosarcoma	Ifosfamide
Radiotherapy after chemotherapy	8		
EBRT ± VB	5		
		Serous adenocarcinoma	Carboplatin
		Carcinosarcoma	Ifosfamide
		Carcinosarcoma	Ifosfamide
		Carcinosarcoma	Ifosfamide
		Carcinosarcoma	Ifosfamide
VB alone	3		
		Serous adenocarcinoma	Carboplatin
		Serous adenocarcinoma	Cisplatin
		Carcinosarcoma	Carboplatin

CCRT, concurrent chemoradiotherapy; EBRT, external beam radiation therapy; n, number; VB, vaginal brachytherapy.
