# Supplementary material for: Impacts of Postoperative Adjuvant Therapies on the Survival of Women with High-Risk Early-Stage Endometrial Cancer: A Cohort Study
Source: Cancers (Basel). 2025 Jan 8;17(2):187. doi: 10.3390/cancers17020187 (PMC11764345; doi:10.3390/cancers17020187)
Supplement: Supplementary file 1 [file cancers-17-00187-s001.zip › Table S3.pdf]

Supplemental Table S3. Distribution of the types of treatments according to the type of histology in women with high-risk EEC.

	Total (n = 1341)	Endometrioid (n = 794)	Non-endometrioid (n = 547)
Primary surgery alone, n	299		299
Primary surgery + adjuvant radiotherapy, n			
EBRT ± VB	515	466	49
VB alone	26		26
Primary surgery + adjuvant chemotherapy, n	392	232	160
Primary surgery + adjuvant chemoradiotherapy, n	18	6	12
Primary surgery + adjuvant hormone therapy, n	91	90	1

EBRT, external beam radiation therapy; n, number; VB, vaginal brachytherapy.
