# Supplementary material for: Impacts of Postoperative Adjuvant Therapies on the Survival of Women with High-Risk Early-Stage Endometrial Cancer: A Cohort Study
Source: Cancers (Basel). 2025 Jan 8;17(2):187. doi: 10.3390/cancers17020187 (PMC11764345; doi:10.3390/cancers17020187)
Supplement: Supplementary file 1 [file cancers-17-00187-s001.zip › Table S4.pdf]

Supplemental Table S4. Distribution of adjuvant radiotherapy and chemoradiotherapy according to the type of histology in women with high-risk EEC.

	Total (n = 559)	Endometrioid (n = 472)	Non-endometrioid (n = 87)
EBRT ± VB, n	515	466	49
VB alone, n	26		26
CCRT, n	2		2
Chemotherapy after radiotherapy, n	8	6	2
Radiotherapy after chemotherapy, n	8		8

EBRT, external beam radiation therapy; n, number; VB, vaginal brachytherapy.
